# Systematic analysis of the ability of Nitric Oxide donors to dislodge biofilms formed by *Salmonella enterica* and *Escherichia coli* O157:H7

**DOI:** 10.1186/s13568-014-0042-y

**Published:** 2014-06-05

**Authors:** Massimiliano Marvasi, Charles Chen, Manuel Carrazana, Ian A Durie, Max Teplitski

**Affiliations:** 1 Soil and Water Science Department, University of Florida-IFAS, Gainesville, FL, USA; 2 Microbiology and Cell Science Department, University of Florida-IFAS, Gainesville, FL, USA; 3 Cancer and Genetics Research Center, Genetics Institute, 2033 Mowry Road, Gainesville 32611, FL, USA

**Keywords:** Biofilm control, Bacterial signaling, Food-borne pathogens, Nitric oxide

## Abstract

Biofilms in the industrial environment could be problematic. Encased in extracellular polymeric substances, pathogens within biofilms are significantly more resistant to chlorine and other disinfectants. Recent studies suggest that compounds capable of manipulating nitric oxide-mediated signaling in bacteria could induce dispersal of sessile bacteria and provide a foundation for novel approaches to controlling biofilms formed by some microorganisms. In this work, we compared the ability of five nitric oxide donors (molsidomine, MAHMA NONOate, diethylamine NONOate, diethylamine NONOate diethylammonium salt, spermine NONOate) to dislodge biofilms formed by non-typhoidal *Salmonella enterica* and pathogenic *E. coli* on plastic and stainless steel surfaces at different temperatures. All five nitric oxide donors induced significant (35-80%) dispersal of biofilms, however, the degree of dispersal and the optimal dispersal conditions varied. MAHMA NONOate and molsidomine were strong dispersants of the *Salmonella* biofilms formed on polystyrene. Importantly, molsidomine induced dispersal of up to 50% of the pre-formed *Salmonella* biofilm at 4°C, suggesting that it could be effective even under refrigerated conditions. Biofilms formed by *E. coli* O157:H7 were also significantly dispersed. Nitric oxide donor molecules were highly active within 6 hours of application. To better understand mode of action of these compounds, we identified *Salmonella* genomic region *recA-hydN*, deletion of which led to an insensitivity to the nitric oxide donors.

## Introduction

Every natural wet surface is a substrate for microbial biofilms. These sessile multicellular microbial consortia are embedded within the self-produced extracellular polymeric substances (EPS) (Costerton et al. [1987]; Marvasi et al. [2010]; Solano et al. [2002]; Wingender et al. [1999]). In food handling facilities, biofilms could be particularly problematic. While pathogens do not typically make up the bulk of the biofilms formed in the industrial settings, *Salmonella spp, Listeria spp,* pathogenic strains of *E. coli, Yersinia spp, Pseudomonas spp, Shigella spp, Staphylococcus spp,* and *Bacillus spp* can be found in biofilms on various contact surfaces (Blanpain-Avet et al. [2011]; Shi and Zhu [2009]). Because microbes in biofilms are significantly more resistant to chlorine and other disinfectants (Zhang and Mah [2008]), there is a pressing need to identify compounds capable of controlling biofilms by other means.

A discovery of the nitric oxide’s function in inducing biofilm dispersal led to concerted efforts on the identification of the compounds capable of dislodging biofilms (Landini et al. [2010]; McDougald et al. [2012]). Several disinfectants, antibiotics and messenger molecules have been studied for their ability to disperse biofilms (Barraud et al. [2006]; Barraud et al. [2012]; Barraud et al. [2009a]; Huynh et al. [2012]; McDougald et al. [2012]). Nitric oxide (NO) gas and NO donors are currently used clinically (Regev-Shoshani et al. [2010]). In bacteria and eukaryotes, nitric oxide is a signaling molecule, active at very low concentrations (Gaupels et al. [2011]; Simontacchi et al. [2013]). At low concentrations, it is effective as a biofilm dispersant, functioning as a messenger rather than a generic poison (Barraud et al. [2006]; Barraud et al. [2009a]). Nitric oxide can be delivered to biofilms using NO donor molecules or impregnated onto nanoparticles (Slomberg et al. [2013]; Wang et al. [2005]). The application of NO donors has the same effect on dispersal of biofilms as the direct addition of nitric oxide and is less toxic than the application of the nitric oxide gas (Barraud et al. [2009a]). Over 105 NO donors have been characterized, but only few of them have been tested for controlling industrial biofilms (Wang et al. [2005]). Among these, activity of sodium nitroprusside has been recently characterized in detail. It can produce a flux of nitric oxide of 30 pmol cm^−2^ s^−1^, and this can efficiently reduce the adhesion of *Staphylococcus aureus*, *Staphylococcus epidermidis*, and *E. coli* by 96%, 48%, and 88%, respectively (Charville et al. [2008]). Therefore, it appears that nitric oxide could have a universal effect on the dispersal of bacterial biofilm including both Gram-positive and Gram-negative bacteria (Xiong and Liu [2010]).

In this work, we tested the effectiveness of NO donors in dispersal of biofilms formed by common foodborne pathogens (non-typhoidal *Salmonella enterica* and enterohaemorrhagic *E. coli*, EHEC) on materials that are common in the food industry. The rationale for this study was based on the reports that the same NO donors have different dispersion potential depending on the bacterial strain, temperature and surface properties (Barraud et al. [2006]; Barraud et al. [2009a]; Charville et al. [2008]; Gilberthorpe and Poole [2008]). Because the ultimate goal of these experiments is to identify commercially available compounds for industrial applications, the following criteria were used to select candidate compounds: 1) low/moderate toxicity; 2) have no more than 0.1% of probable, possible or confirmed human carcinogenicity according to the International Agency for Research on Cancer (IARC); 3) low/moderate cost; 4) commercially availability. Based on these criteria, the following compounds were selected: molsidomine (N- (ethoxycarbonyl)- 3- (4- morpholinyl)- sydnone imine), MAHMA NONOate (6-(2-Hydroxy-1-methyl-2-nitrosohydrazino)-N-methyl-1-hexanamine), spermine NONOate (N-[4-[1-(3-Aminopropyl)-2-hydroxy-2-nitrosohydrazino]butyl]-1,3-propanediamine), diethylamine NONOate diethylammonium salt, and diethylamine NONOate sodium.

The mechanisms by which NO effects the transition from sessile biofilm organisms to free-swimming bacteria are not entirely clear (Barraud et al. [2006]; Barraud et al. [2009a]).

Microarray studies have revealed that *P. aeruginosa* genes involved in adherence are down regulated upon exposure to nitric oxide (Firoved et al. [2004]), and the involvement of NO in regulating biofilm formation and dispersal in *P. aeruginosa* was also supported by several studies (Barraud et al. [2006]; Barraud et al. [2009a]; Darling and Evans [2003]; Van Alst et al. [2007]). Several genes involved in the production and perception of this signaling molecule have been characterized in *Pseudomonas aeruginosa* PAO1. The chemotaxis protein BdlA is involved in biofilm dispersion of *P. aeruginosa*: biofilms formed by the *bdlA* mutant do not detach when exposed to low doses of NO in continuous-flow cultures (Barraud et al. [2009a]; Petrova and Sauer [2012]). *P. aeruginosa nirS* and *norCB* encode a NO_2_^−^ reductase and NO reductase. A mutation in *nirS* leads to a reduced biofilm dispersion (Barraud et al. [2006]), while biofilms formed by a NO reductase-deficient strain Δ*norCB* did not shift to the planktonic state when exposed to endogenous nitric oxide. No homologs of NirS, NorCB are found in *Salmonella*. However, when grown anaerobically with nitrate, *Salmonella* is capable of generating NO after nitrite addition, likely via products of *fnr* and *hmp* genes (Gilberthorpe and Poole [2008]). Because the mechanisms of nitric oxide-mediated signaling in *Salmonella* appear to be distinct from those in *P. aeruginosa*, this study also attempted to elucidate genes that are potentially involved in these signaling pathways and contribute to biofilm dispersal in *Salmonella.*

## Materials and methods

### Bacterial strains and culture media

*Escherichia coli* EHEC O157:H7 ATCC 43888, *Salmonella enterica* serotovar Typhimurium ATCC14028*,* sv. Braenderup 04E01347, Braenderup 04E01556, Braenderup 04E00783, sv. Montevideo LJH519, sv. Javiana ATCC BAA-1593 and sv. Newport C6.3 (Noel et al. [2010]) were used in this study. When a cocktail of *Salmonella* strains was used, it was a mix of equal volumes of six strains: three strains of the serovar Braenderup (04E01347, 04E01556, 04E00783), sv. Montevideo LJH519, Javiana (ATCC BAA-1593) and Newport (C6.3). pGFP-ON (a strongly fluorescent construct carrying GFP protein expressed from the *Salmonella dppA* promoter (Noel et al. [2010])) was transformed into the strains of interest by electroporation.

*S.* Typhimurium A9 is derived from *S.* Typhimurium ATCC14028 and lacks the genomic region between 2,974,854 and 2,990,668 nt of NC_003197.1 (*recA* through *hydN*), which was replaced with a kanamycin-resistance cassette. It was constructed by sequential Datsenko and Wanner mutagenesis as in (Santiviago et al. [2009]).

All strains were maintained as frozen glycerol stocks, and were sub-cultured into Luria Bertani medium with appropriate antibiotics (50 μg mL^−1^ kanamycin, 100 μg mL^−1^ ampicillin).

### Nitric Oxide donors

All were purchased from Sigma Aldrich (St. Luois, MO, USA). For each compound, 1 mmol L^−1^ stock solutions were prepared in phosphate-buffered saline, pH 7.3 (PBS, Fisher, Waltham, MA, USA) and aliquots were stored at −80°C. For the essays, serial dilutions were always prepared fresh in ice-cold PBS just before the experiments and used within 5 minutes of their preparation. The biofilm dispersion potential of the five molecules was tested on polystyrene and polypropylene. The ability of molsidomine to disperse biofilms was also tested on stainless steel.

### Biofilm formation and dispersal on plastics

Overnight Luria Bertani cultures of *Salmonella* or *E. coli* strains were diluted 1:100 in CFA medium as described previously (Teplitski et al. [2006]), and 100 μL of the diluted cultures were aliquoted into wells of 96-well polypropylene and polystyrene plates (Fisher, Waltham, MA, USA). Plates with bacteria were incubated for 24 hours at 37°C inside a Ziploc bag. Upon completion of the incubation, the medium with planktonic bacteria was removed and serial dilutions of nitric oxide donors in PBS (in 200 μL) were added to the wells with biofilms. Dispersal experiments were conducted at 22°C or 4°C for 6 and 24 hours. Dispersal was measured by staining the remaining biofilms with 1% crystal violet in ethanol, as described previously (Merritt et al. [2005]; O’Toole and Kolter [1998]).

In parallel, to validate the staining approach, detachment of cells from biofilms was also measured by directly monitoring the increase of fluorescence of planktonic cells of *S.* Typhimurium 14028 pGFP-ON using Victor-2 multimode plate reader with a 485 nm/535 nm excitation/emission filter (Perkin Elmer, Waltham, MA, USA).

### Luminescence tests

Effects of selected NO donors on light production by a constitutively luminescent *Salmonella* strain were characterized as indirect assessments of toxicity of the compounds. Two hundred microliters of Luria Bertani broth inoculated with the overnight, 1:50 diluted culture of S. Typhimurium 14028 pTIM2442 (harboring the *luxCDABE* driven by a strong constitutive phage λ promoter*,* Alagely et al., [2011]) were grown in black polystyrene plates (Corning, New York, USA) in presence of serial dilutions of Molsidomine. Molsidomine was diluted in PBS (9.89gL^−1^) (Fisher Scientific, Waltham, MA, USA) to final concentrations of 10μmolL^−1^, 10nmolL^−1^, and 10pmolL^−1^. PBS was used as a control. Luminescence of *S.* Typhimurium 14028 pTIM2442 was measured over time using Victor-2 multimode plate reader (Perkin Elmer, Waltham, MA, USA). Each experiment included 12 replicas.

### Biofilm formation on stainless steel

Biofilms were formed on the stainless steel culture tube closures (Fisher Scientific, Waltham, MA, USA) essentially as described above for plastics with the following modifications: only dilutions of molsidomine were used, and only 24 hours of contact time was tested. Biofilm dispersal was tested by monitoring fluorescence under a multimode microplate reader equipped with a 485 nm/535 nm excitation/emission filter (Perkin Elmer, Waltham, MA, USA).

### Additive effect of the disinfectant SaniDate 12.0 with nitric oxide donors

Biofilms of *S.* Typhimurium ATCC14028 were set up as above using overnight cultures of the pathogen diluted 1:100 in the CFA medium in wells of 96-well polypropylene plates (Fisher, Waltham, MA, USA). Plates with bacteria were incubated for 24 hours at 37°C inside a Ziploc bag. Upon completion of the incubation, the medium with planktonic bacteria was removed and 10nmolL^−1^ of Molsidomine or MAHMA nonoate were added to the wells with biofilms. As controls, BPS alone was used. Plates were incubated at 22°C for 24 hours. Upon completion of the incubation, planktonic cells were removed, wells were washed twice with PBS and 200 μL of SaniDade 12.0 (BioSafe System, Hartford, CT, USA) diluted as per manufacturer’s recommendations and were loaded into the wells. The disinfectant was incubated for 10 minutes, after the incubation time, biofilm dispersal was measured by staining the remaining biofilms with 1% crystal violet in ethanol, as described previously (Merritt et al. [2005]; O’Toole and Kolter [1998]). 12 replicas for each experiment were done.

### Statistical analysis

The statistical software JMP (SAS) package was used to infer the One-way ANOVA analysis (p < 0.05). Tukey means separation analysis was inferred in order to group the means.

## Results

### Biofilm formation on different plastics

More robust biofilms were formed by *Salmonella* and *E. coli* strains on polypropylene than on polystyrene (Additional file 1: Figure S1). On polypropylene and polystyrene, *S. enterica* sv Typhimurium 14028 formed more biofilms under these conditions than *E. coli* O157:H7 (Additional file 1: Figure S1). Biofilm formation is known to vary depending on the surface, the media used to develop biofilms, as well as the protocol adopted (Kroupitski et al. [2009]; Teplitski et al. [2006]).

### Biofilm dispersal by molsidomine

**Figure 1:**
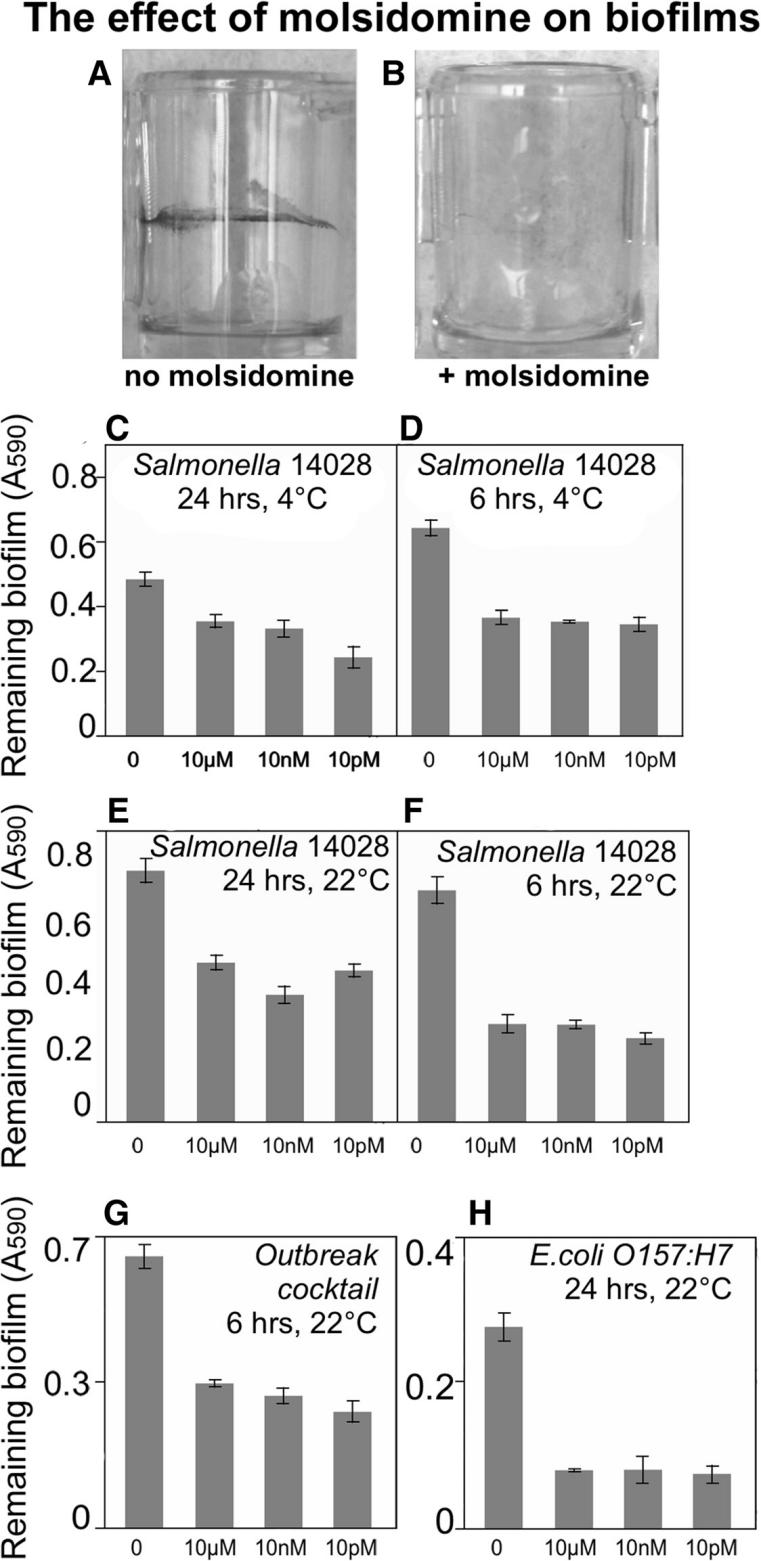
**Biofilm dispersal by molsidomine.** Exposure to molsidomine doses ranging from 10 μM to 10pM induced biofilm dispersal as measured by staining of the attached cells with crystal violet. “0” indicates control, an existing biofilm not treated with molsidomine. **A.** Biofilm formed by *S. enterica* sv. Typhimurium 14028 in wells of a microtiter plate before treatment with molsidomine. Attached cells are visualized with crystal violet staining. **B.** Residual biofilm after treatment with 10 nM of molsidomine. **C**→**H.** Induction of biofilm dispersal by molsidomine. Biofilms were pre-formed on polystyrene. Contact time and temperature at which biofilms were exposed to the chemical are listed above each panel. Concentrations of molsidomine are indicated on the x-axis. Error bars are standard errors.

Molsidomine releases nitric oxide and forms polar metabolites rapidly; its half-life is 1 to 2 hours in plasma at pH 7.4 (Rosenkranz et al. [1996]). In terms of biofilm dispersal, molsidomine was the most potent molecule. It was effective in dislodging biofilms formed by *S. enterica* sv Typhimurium 14028, the cocktail of the six *Salmonella* outbreak strains and *E. coli* O157:H7. Molsidomine was particularly effective on polypropylene (Figure 1). It was most effective at 22°C, inducing dispersal of ~50% of biofilms formed by *Salmonella* 14028 and the cocktail of six *Salmonella* outbreak strains after incubation for 6 hours (Figure 1, p < 0.0001). Up to 75% of biofilm dispersal was observed after a 24-hrs treatment of *E. coli* O157:H7 biofilms with molsidomine (Figure 1, p < 0.0001). Biofilm dispersal by molsidomine was also observed on polystyrene (Additional file 1: Table S1). *Salmonella* biofilms preformed in polystyrene wells were dislodged after 24 hours of contact time at room temperature. Molsidomine was able to induce some dispersal even when biofilms were treated with the compound at 4°C. Intriguingly, the strongest dispersal was observed in response to the treatment with 10 picomolar concentrations of molsidomine (Figure 1). Such potency of the compound at very low concentrations makes it a potentially interesting candidate for commercial applications.

To test whether the decrease in the staining of the attached cells was caused by an increase in the number of planktonic cells, biofilms formed by fluorescent *Salmonella* 14028 pGFP-ON on polypropylene were treated with molsidomine. Fluorescence of the planktonic and attached cells was measured after 0, 3 and 6 hours of exposure to the nitric oxide donor. The increase in total fluorescence of planktonic cells upon treatment of biofilms with molsidomine was statistically significant (Additional file 1: Table S1), reflecting an increase in detachment at the tested concentrations. Similarly, molsidomine treatment of the *Salmonella* biofilms formed on stainless steel resulted in ~0.3 log increase in fluorescence of the planktonic cells (data not shown).

The ability of the constitutively luminescent *Salmonella* construct driven by a phage λ promoter to produce light in the presence of molsidomine was used as an indirect assessment of the toxicity of the compound, and its ability to generally disrupt metabolism or respiration of the bacteria. As shown in Additional file 1: Figure S2, even though modest decrease in luminescence was observed in molsidomine-treated cultures, the reduction of luminescence was not greater than 1-10% of the control.

### Dose-dependent biofilm dispersal by diethylamine NONOate diethylammonium

**Figure 2:**
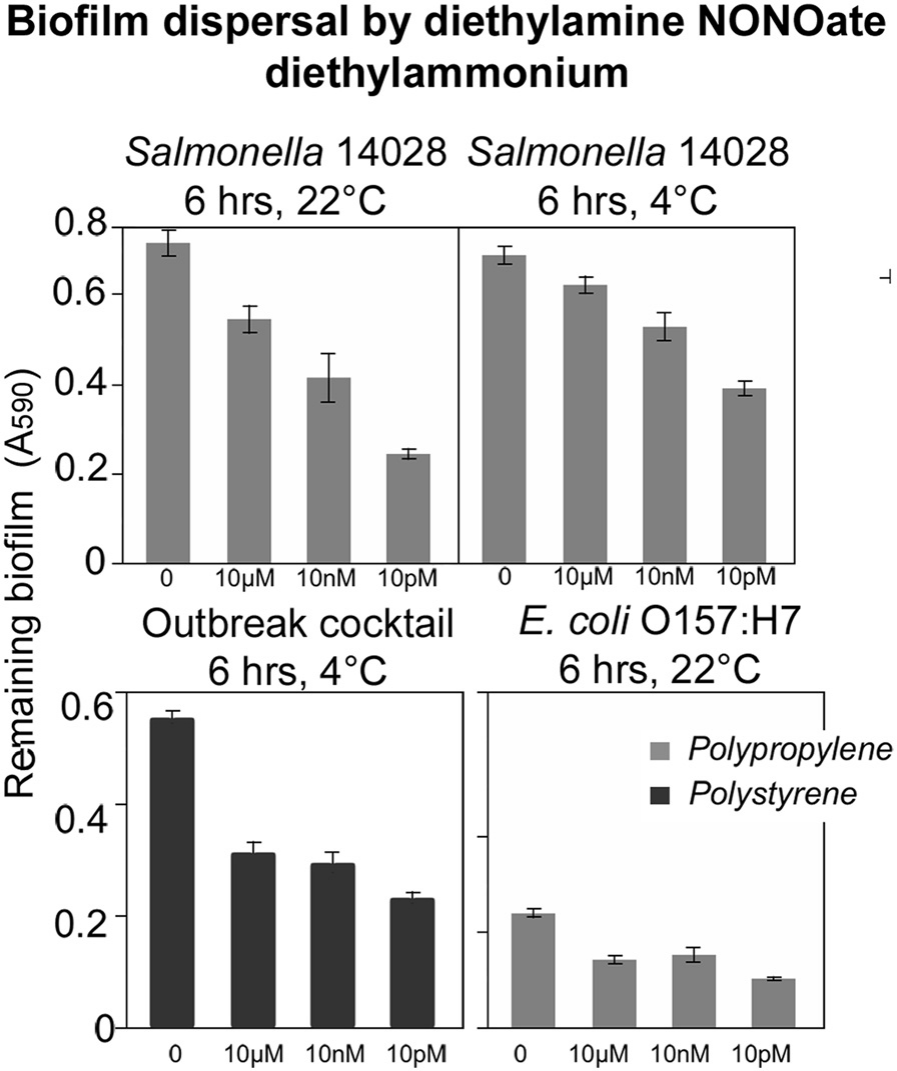
**The effect of diethylamine NONOate diethylammonium on biofilms.** Remaining biofilms were measured by crystal violet staining, which was solubilized in diluted acetic acid and the absorbance at 590 nm was measured. Concentrations of the nitric oxide donor are on the x-axis. Contact time and temperature at which pre-formed biofilms were treated with diethylamine NONOate diethylammonium are indicated above each panel.

Diethylamine NONOate diethylammonium represents a class of molecules which spontaneously dissociate in a pH-dependent manner, with a half-life of 16 minutes at 22-25°C, pH 7.4 to liberate 1.5 moles of NO per mole of the parental compound (Keefer et al. [1996]; Maragos et al. [1991]). Biofilm dispersal in response to diethylamine NONOate diethylammonium was dose-dependent (Figure 2), however the relationship was inverse: low concentrations of the nitric oxide were associated with higher biofilm dispersion. The strong activity in the picomolar range is similar to what was observed with molsidomine.

Diethylamine NONOate diethylammonium was most potent at dispersing biofilms on polypropylene following a 6-hours incubation. A strong dispersal of biofilms formed by *Salmonella* ATCC 14028 on polypropylene was observed (Figure 2). Biofilms formed by *E. coli* O157:H7 were also dispersed by diethylamine NONOate diethylammonium, but the dispersal was lower when compared with *Salmonella*. The biofilm formed by the cocktail of six *Salmonella* outbreak strains formed on polystyrene was also dispersed (Figure 2 and Additional file 1: Table S2 and Additional file 1: Table S3).

### The effect of diethylamine NONOate sodium salt hydrate, MAHMA NONOate and spermine NONOate on biofilms

**Figure 3:**
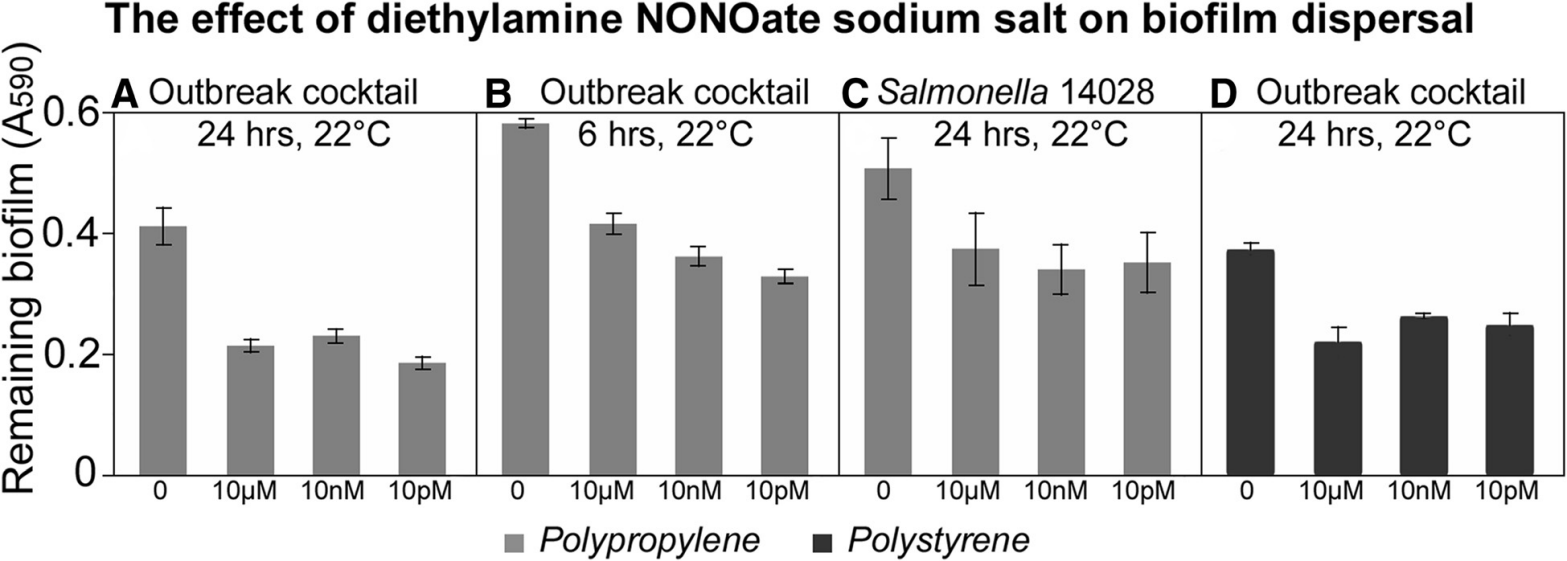
**Dispersal of** ***Salmonella*** **biofilms by diethylamine NONOate sodium salt.** Biofilms were formed overnight by either *S. enterica* sv. Typhimurium ATCC14028 or a cocktail of six *Salmonella enterica* strains (sv. Braenderup 04E01347, Braenderup 04E01556, Braenderup 04E00783, sv. Montevideo LJH519, sv. Javiana ATCC BAA-1593 and sv. Newport C6.3) linked to produce-associated outbreaks of gastroenteritis. Biofilms were pre-formed on either polystyrene (black bars) or polypropylene (grey bars) prior to the treatment with diethylamine NONOate sodium salt. Concentrations of the nitric oxide donor are on the x-axis. Contact time and temperature at which the treatment took place are indicated above each panel. Residual biofilms were quantified by staining with crystal violet. Error bars are standard errors.

Of the tested compounds, diethylamine NONOate sodium salt was the least effective biofilm dispersant following 24 and 6 hours incubation. The strongest dispersion was observed in biofilms formed on polypropylene by the cocktail of *Salmonella* strains and *Salmonella* Typhimurium ATCC 14028 (Figure 3). Up to 50% of reduction of biofilm was detected at room temperature for *Salmonella* 14028 and the outbreak strains on polystyrene (Figure 3). On polystyrene, biofilm formation by the cocktail of the *Salmonella* strains was reduced by up to 30% when compared with the control. Diethylamine NONOate was also active in dispersing biofilms formed by *E. coli* O157:H7, even though the dispersion was very limited (~20% when compared with the control) (Additional file 1: Table S2 and Additional file 1: Table S3).

The effectiveness of MAHMA NONOate (which has half-life of minutes) was compared to that of sperimne NONOate, which has half-life of several hours. This affects potency and sustainability of the treatment, for MAHMA NONOate showing up to 40 times the potency of spermine NONOate, but the latter showing activaty for hours, compared with few minutes for MAHMA NONOate (Wang et al. [2005]). MAHMA NONOate is also an optimal dispersant of preformed biofilm of *Pseudomonas aerouginosa* biofilm (Barnes et al. [2013]). Therefore, the two molecules were compared within 6 hours of incubation with *Salmonella* and *E. coli* biofilms. As expected MAHMA NONOate was a more effective dispersant on both the materials (Table 1). However, spermine NONOate was mainly effective at releasing biofilms formed on polypropylene (Table 1).

**Table 1:** The effect of Spermine NONOate and MAHMA NONOate on preformed biofilm

			Probability > F (p = 0.05)*	
**Material**	**Strain**	**Incubation temperature**	**Spermine NONOate**	**MAHMA NONOate**
Polypropylene	*Salmonella* 14028	4°C	n.s.	n.s.
Polypropylene	*Salmonella* 14028	22°C	n.s.	0.0049
Polypropylene	*E.coli* O157:H7	4°C	0.0273	n.s.
Polypropylene	*E.coli* O157:H7	22°C	0.0486	<.0001
Polypropylene	*Salmonella* cocktail	4°C	0.0036	0.0185
Polypropylene	*Salmonella* cocktail	22°C	0.0003	<.0001
Polystyrene	*Salmonella* 14028	4°C	0.0418	0.0229
Polystyrene	*Salmonella* 14028	22°C	n.s.	<.0001
Polystyrene	*E.coli* O157:H7	4°C	n.s.	n.s.
Polystyrene	*E.coli* O157:H7	22°C	n.s.	n.s.
Polystyrene	*Salmonella* cocktail	4°C	n.s.	0.0184
Polystyrene	*Salmonella* cocktail	22°C	0.0183	<.0001

*Statistically significant effects (p < 0.05) of the NO donor treatments when compared with the untreated control; “n.s.” not statistically significant biofilm dispersion.

**Figure 4:**
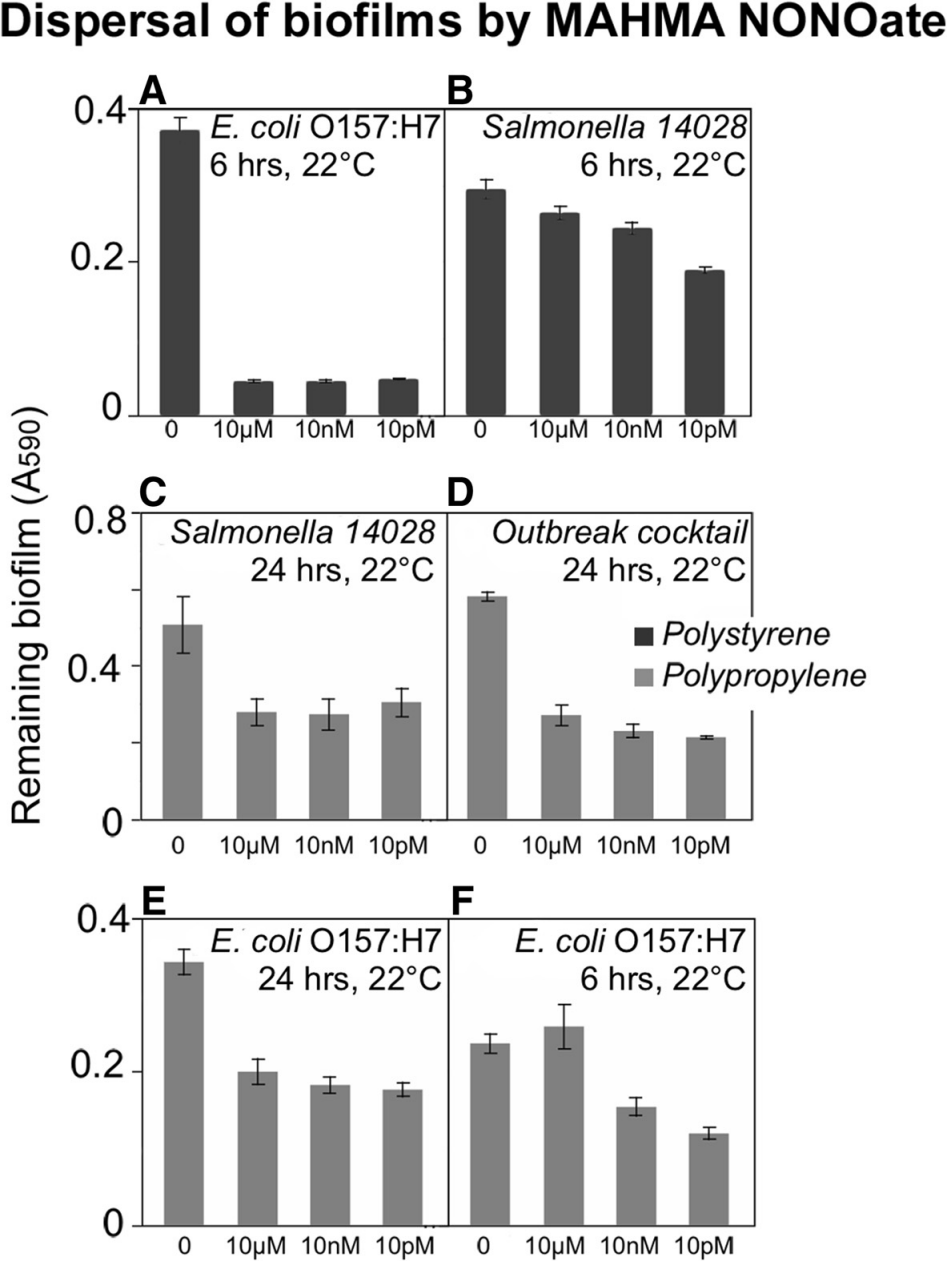
**Biofilm dispersal by MAHMA NONOate.** Biofilms were formed by *E. coli* O157:H7, *Salmonella* sv. Typhimurium 14028 and a cocktail of the six *Salmonella* strains linked to human produce-related gastroenteritis outbreaks on polystyrene (black bars) or polypropylene (grey bars) prior to the treatment with MAHMA NONOate. Contact times and temperatures at which biofilms were exposed to the nitric oxide donor are listed above each panel. Concentrations of the nitric oxide donor are on the x-axis.

**Figure 5:**
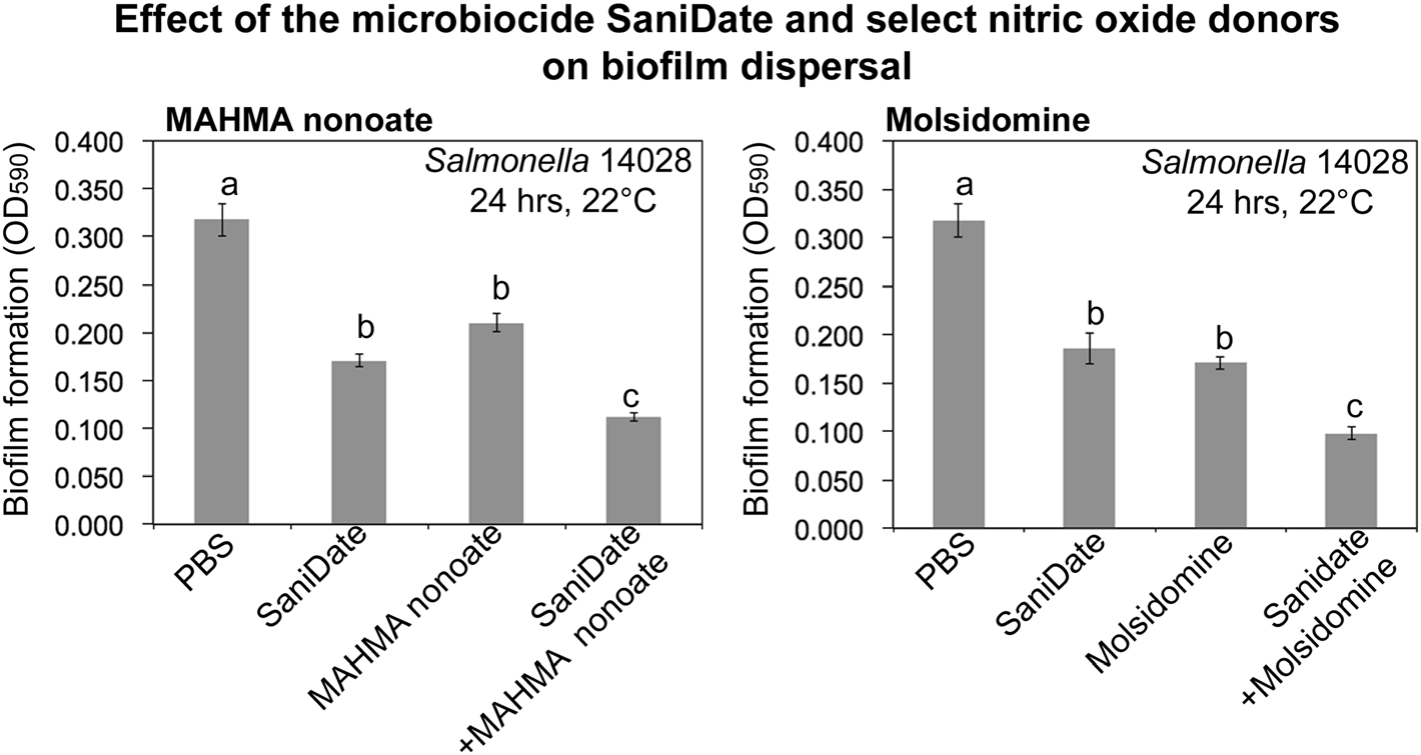
**Additive effect of the microbiocide SaniDate and nanomolar concentrations of Molsidomine and MAHMA nonotate.** Bars represent the standard error. Different letters represent significant different means (p = 0.05).

MAHMA NONOate also appears to be a broadly active molecule in dispersing biofilm formed both on polypropylene and polystyrene (Figure 4): it released up to 70% of *E. coli* biofilms pre-formed on polystyrene at room temperature after a 6-hour treatment (Figure 4). Biofilms formed by *S. enterica* sv Typhimurium ATCC14028 were also dispersed by MAHMA NONOate at room temperature, but compared to spermine NONOate, it was more effective on polystyrene (Figure 4). On polypropylene, up to 50% of *S. enterica* sv Typhimurium ATCC14028 and the cocktail of six *Salmonella* outbreak strains biofilms were dispersed by MAHMA NONOate when incubated for 24 hours at room temperature (Figure 4). Biofilms formed by *E. coli* O157:H7 were also effectively dispersed on polypropylene but less when compared with polystyrene (Figure 4). Effectiveness of MAHMA nonoate in dispersing biofilms was further increased by treatment with the microbiocide SaniDate (Figure 5).

### The recA-hydN genomic region is involved in Salmonella biofilm dispersal

**Figure 6:**
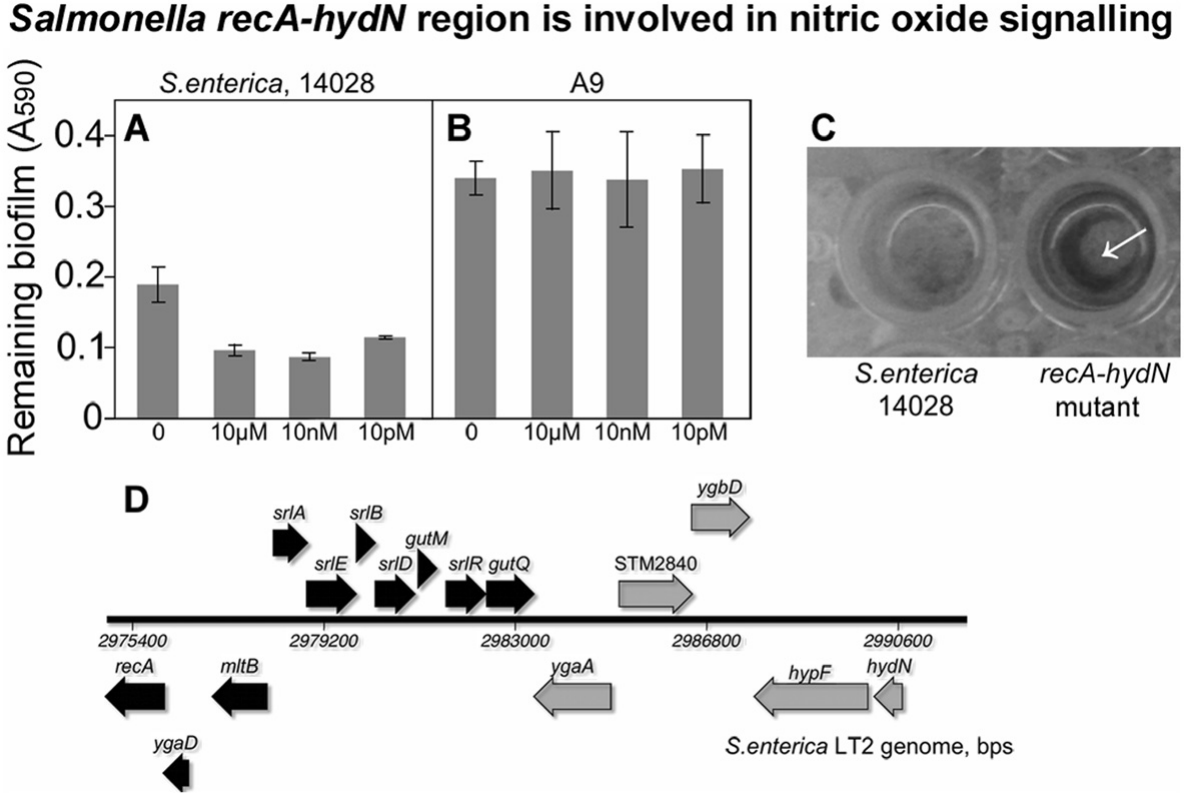
**Involvement of the** ***Salmonella recA-hydN*** **genomic region in the nitric oxide-mediated signaling.***Salmonella* sv. Typhimurium A9 (mutant lacking a 15kB fragment spanning *recA-hydN* genes) formed more abundant biofilms, which were not responsive to treatment with nitric oxide donor molsidomine. Detachment of the wild type biofilms in response to molsidomine **(A)**. Lack of response to molsidomine by the A9 mutant **(B)**. Appearance of the biofilms formed by the A9 mutant and the wild type **(C)**. Genomic context of the A9 deletion **(D)**. Thick arrows are annotated ORF. Genes with putative functions in nitric oxide signaling are shaded grey.

In order to better understand how nitric oxide donors induce dispersal of *Salmonella* biofilms, the genome of *S.* Typhimurium 14028 was scanned for the homologs of known genes involved in nitric oxide signaling. A mutant lacking the ~15kB *Salmonella enterica* sv Typhimurium ATCC14028 genomic region spanning 15 genes (*recA*-*hydN*) was studied (Figure 6). This region includes putative NO-reductase machinery: *ygaA*, an anaerobic nitric oxide reductase transcriptional regulator; STM2840, an anaerobic nitric oxide reductase flavorubredoxin; *ygbD*, nitric oxide reductase; *hypF*, hydrogenase maturation protein; *hydN*, an electron transport protein HydN. Proteins encoded region can be involved in transferring electron to NO, its detoxification and generation of nitrous oxide. Consistent with the predicted functions of the deleted genes, the Δ*recA*-*hydN* mutant formed a more robust biofilm on polystyrene compared to the wild type, and did not respond to the 24-hrs treatment with molsidomine (Figure 6).

## Discussion

Controlling biofilms on surfaces of clinical and industrial importance has emerged as an important goal. While an impressive toolbox is potentially available to those aiming to prevent microbes from attaching to surfaces and forming biofilms (Campoccia et al. [2013]; Chen et al. [2013]), the approaches for controlling existing biofilms are significantly more limited. A seminal discovery that self-produced nitric oxide acts as a dispersal cue for *Pseudomonas aeruginosa* biofilms (Barraud et al. [2006]) sparked interest in exploring the use of the NO gas, molecules and nanoparticles capable of releasing it (Landini et al. [2010]; McDougald et al. [2012]). Various nitric oxide generating molecules and nanoparticles can be used effectively to dislodge biofilms formed by Gram-negative *P. aeruginosa* and *E. coli*, Gram-Positive *Staphylococcus aureus* and *S. epidermidis* (Barraud et al. [2009b]; Cherayil and Antos [2001]; Slomberg et al. [2013]) and prevent attachment of zoospores of the green algae *Ulva* (Thompson et al. [2010]). However, in some bacteria (*Shewanella oneidensis, Vibrio harveyi*), perception of NO leads to an increased biofilm formation (Landini et al. [2010]; Plate and Marletta [2012]). Therefore, even though NO is a signal employed by a diversity of organisms, responses to it are not universally conserved. Because of these biological differences in the consequences of NO detection, it is important to establish how and to what extent commercially available nitric oxide donors can be used for controlling biofilms formed by important foodborne bacterial pathogens, known to persist as biofilms in the industrial facilities.

With this study, we focused on the effects of off-the-shelf NO donors on the biofilms formed by seven strains of *S. enterica* and pathogenic *E. coli* on surfaces that mimic those found in food processing facilities. The five compounds selected for these tests based on their low potential toxicity, commercial availability and predicted potency demonstrated varying levels of specificity and efficacy. Of the compounds tested, molsidomine and diethylamine NONOate diethylammonium are the most promising as they are capable of dislodging biofilms formed by *S*. Typhimurium 14028, a cocktail of six *Salmonella* strains isolated from outbreaks and *E. coli* O157:H7 under most of the tested conditions. For most of the compounds tested, their highest activity appears to be in the picomolar range, suggesting that their applications could be further optimized for the economical and effective industrial applications. The activity of the compounds in the nano- and picomolar ranges further suggests that NO acts as a potent cue, consistent with previous reports. Low biofilm-dispersing activity of the nitric oxide donors at higher concentrations is, perhaps, not surprising considering that NO can be bactericidal at a concentration of mg L^−1^ (McDougald et al. [2012]; Miller et al. [2009]), and the methods employed in this study would not necessarily distinguish between live and dead bacteria within an existing biofilm.

Interestingly, some compounds (molsidomine or diethylamine NONOate diethylammonium) were effective at dispersing biofilms under refrigerated conditions (4°C). Temperature is an important factor affecting the dissociation constant. NONOate (s) are very sensitive to temperature: a 1°C change from 37°C can results in an approximate 13% change in NO release (Ramamurthi and Lewis [1997]). Therefore, it is reasonable to hypothesize that when biofilms are exposed to the nitric oxide donors at lower temperatures, they experienced the treatment for a longer period of time, and this increased their potency. The implications of this observation for industrial applications are potentially exciting: the ability of the nitric oxide donors to disperse biofilms at 4°C makes them good candidates for cleaning refrigerated surfaces, common in the food industry and even removing pathogen biofilms from refrigerated foods. While *Salmonella* and *E. coli* are thought to be metabolically inactive under these conditions, their populations in biofilms formed on refrigerated foods remain relatively steady over an extended period of time (Kroupitski et al. [2009]). Furthermore, a complement of *Salmonella* genes that were differentially expressed during biofilm formation on cut lettuce during cold storage has been identified (Kroupitski et al. [2013]), suggesting that even though the cells of these human pathogens are not dividing, physiological processes and gene expression still take place under refrigerated conditions. It would be of great interest to determine whether these specific genes induced in biofilms on foods at 4-8°C could be subject to manipulation by NO. More broadly, it remains to be determined which of the *Salmonella* genes involved in biofilm formation (Hamilton et al. [2009]; Teplitski et al. [2006]) are subject to regulation by nitric oxide, and – conversely – which of the known *Salmonella* genes responsive to nitric oxide contribute to the NO-mediated biofilm dispersal (Henard and Vazquez-Torres [2011]; Karlinsey et al. [2012]; Richardson et al. [2011]).

## Competing interests

This research was supported by funding provided by the Florida Tomato Committee, Grant #106486 and by the UC-Davis Center for Produce Safety, Grant #2014-308.

## Authors’ contributions

MM, MT conceived experiments; MM, CC, MC, IAD conducted experiments; MM analyzed data; MM, MT wrote the manuscript. All authors read and approved the final manuscript.

## Additional file

## Supplementary Material

Additional file 1: Figure S1. Biofilm formation on different plastics by *Salmonella* and *E. coli* O157:H7. Biofilms were established on polypropylene and polystyrene surfaces in CFA medium for 24 hrs and stained with 1% crystal violet and then washed. The absorbed crystal violet and the biofilm were dissolved in 33% acetic acid and A590 was measured with a spectrophotometer. Error bars represent standard error. Significant different means are displayed with different letters. **Figure S2.** Luminescence of *Salmonella* 14028 pTIM2442 upon exposure to molsidomine. General metabolic state of the cells was assessed using the redox-coupled FMNH2/Luciferase produced by a *S.* Typhimurium ATCC14028 strain harboring high copy number plasmid in which the *luxCDABE* operon is under the phage λ promoter (pTIM2442). Concentrations of molsidomine to which cultures of *Salmonella* 14028 pTIM2442 were exposed are listed on the figure. Error bars represent the standard error of 12 replicas. **Table S1.** Fluorescence of the *Salmonella* cells detached by molsidomine treatment Measurement of *Salmonella* 14028 planktonic cells detached from preformed biofilm during treatment with molsidomine. polypropylene, *Statistically significant effects (p<0.05) of molsidomine treatment. **Table S2.** The complete set of experiments on the effect of the NO donors on preformed biofilm after 24 hours of contact time. *Statistically significant effects (p<0.05) of the NO donor treatments. n.s., not statistically significant biofilm dispersion. MOL, Molsidomine; M1555, MAHMA NONOate; S150, SPERMINE NONOate; D5431, diethylamine NONOate diethylammonium salt; D184, diethylamine NONOate sodium; S8432 sulfo NONOate disodium salt. **Table S3.** The complete set of experiments on the effect of the NO donors on preformed biofilm after 6 hours of contact time. *Statistically significant effects (p<0.05) of the NO donor treatments. n.s., not statistically significant biofilm dispersion. MOL, Molsidomine; M1555, MAHMA NONOate; S150, SPERMINE NONOate; D5431, diethylamine NONOate diethylammonium salt; D184, diethylamine NONOate sodium; S8432 sulfo NONOate disodium salt. Click here for file

## References

[B1] Alagely A Rajamani S Teplitski M Luminescent reporters and their applications for the characterization of signals and signal-mimics that alter LasR-mediated quorum sensing Methods Mol Biol 2011 4 113 130 10.1007/978-1-60761-971-0_9 21031308

[B2] Barnes RJ Bandi RR Wong WS Barraud N McDougald D Fane A Kjelleberg S Rice SA Optimal dosing regimen of nitric oxide donor compounds for the reduction of *Pseudomonas aeruginosa* biofilm and isolates from wastewater membranes Biofouling 2013 4 2 203 212 10.1080/08927014.2012.760069 23368407

[B3] Barraud N Hassett DJ Hwang SH Rice SA Kjelleberg S Webb JS Involvement of nitric oxide in biofilm dispersal of *Pseudomonas aeruginosa* J Bacteriol 2006 4 21 7344 7353 10.1128/JB.00779-06 17050922 PMC1636254

[B4] Barraud N Schleheck D Klebensberger J Webb JS Hassett DJ Rice SA Kjelleberg S Nitric oxide signaling in *Pseudomonas aeruginosa* biofilms mediates phosphodiesterase activity, decreased cyclic di-GMP levels, and enhanced dispersal J Bacteriol 2009 4 23 7333 7342 10.1128/JB.00975-09 19801410 PMC2786556

[B5] Barraud N Storey MV Moore ZP Webb JS Rice SA Kjelleberg S Nitric oxide-mediated dispersal in single- and multi-species biofilms of clinically and industrially relevant microorganisms Microb Biotechnol 2009 4 3 370 378 10.1111/j.1751-7915.2009.00098.x 21261931 PMC3815757

[B6] Barraud N Kardak BG Yepuri NR Howlin RP Webb JS Faust SN Kjelleberg S Rice SA Kelso MJ Cephalosporin-3′-diazeniumdiolates: targeted NO-donor prodrugs for dispersing bacterial biofilms Angew Chem Int Ed Engl 2012 4 36 9057 9060 10.1002/anie.201202414 22890975

[B7] Blanpain-Avet P Faille C Delaplace G Bénézech T Cell adhesion and related fouling mechanism on a tubular ceramic microfiltration membrane using *Bacillus cereus* spores J Membrane Sci 2011 4 0 200 216 doi:http://dx.doi.org/10.1016/j.memsci.2011.09.041 10.1016/j.memsci.2011.09.041

[B8] Campoccia D Montanaro L Arciola CR A review of the biomaterials technologies for infection-resistant surfaces Biomaterials 2013 4 34 8533 8554 10.1016/j.biomaterials.2013.07.089 23953781

[B9] Charville GW Hetrick EM Geer CB Schoenfisch MH Reduced bacterial adhesion to fibrinogen-coated substrates via nitric oxide release Biomaterials 2008 4 30 4039 4044 10.1016/j.biomaterials.2008.07.005 18657857 PMC2582185

[B10] Chen M Yu Q Sun H Novel strategies for the prevention and treatment of biofilm related infections Int J Mol Sci 2013 4 9 18488 18501 10.3390/ijms140918488 24018891 PMC3794791

[B11] Cherayil BJ Antos D Inducible nitric oxide synthase and *Salmonella* infection Microbes Infect 2001 4 9 771 776 10.1016/S1286-4579(01)01428-9 11489426

[B12] Costerton JW Cheng KJ Geesey GG Ladd TI Nickel JC Dasgupta M Marrie TJ Bacterial biofilms in nature and disease Annu Rev Microbiol 1987 4 435 464 10.1146/annurev.mi.41.100187.002251 3318676

[B13] Darling KE Evans TJ Effects of nitric oxide on *Pseudomonas aeruginosa* infection of epithelial cells from a human respiratory cell line derived from a patient with cystic fibrosis Infect Immun 2003 4 5 2341 2349 10.1128/IAI.71.5.2341-2349.2003 12704103 PMC153226

[B14] Firoved AM Wood SR Ornatowski W Deretic V Timmins GS Microarray analysis and functional characterization of the nitrosative stress response in nonmucoid and mucoid *Pseudomonas aeruginosa* J Bacteriol 2004 4 12 4046 4050 10.1128/JB.186.12.4046-4050.2004 15175322 PMC419947

[B15] Gaupels F Kuruthukulangarakoola GT Durner J Upstream and downstream signals of nitric oxide in pathogen defence Curr Opin Plant Biol 2011 4 6 707 714 10.1016/j.pbi.2011.07.005 21816662

[B16] Gilberthorpe NJ Poole RK Nitric oxide homeostasis in *Salmonella typhimurium*: roles of respiratory nitrate reductase and flavohemoglobin J Biol Chem 2008 4 17 11146 11154 10.1074/jbc.M708019200 18285340

[B17] Hamilton S Bongaerts RJ Mulholland F Cochrane B Porter J Lucchini S Lappin-Scott HM Hinton JC The transcriptional programme of *Salmonella enterica* serovar Typhimurium reveals a key role for tryptophan metabolism in biofilms BMC Genomics 2009 4 599 10.1186/1471-2164-10-599 20003355 PMC2805695

[B18] Henard CA Vazquez-Torres A Nitric oxide and *Salmonella* pathogenesis Front Microbiol 2011 4 84 10.3389/fmicb.2011.00084 21833325 PMC3153045

[B19] Huynh TT McDougald D Klebensberger J Al Qarni B Barraud N Rice SA Kjelleberg S Schleheck D Glucose starvation-induced dispersal of *Pseudomonas aeruginosa* biofilms is cAMP and energy dependent PLoS One 2012 4 8 e42874 10.1371/journal.pone.0042874 22905180 PMC3419228

[B20] Karlinsey JE Bang IS Becker LA Frawley ER Porwollik S Robbins HF Thomas VC Urbano R McClelland M Fang FC The NsrR regulon in nitrosative stress resistance of *Salmonella enterica* serovar Typhimurium Mol Microbiol 2012 4 6 1179 1193 10.1111/j.1365-2958.2012.08167.x 22831173 PMC3438343

[B21] Keefer LK Nims RW Davies KM Wink DA Lester P “NONOates” (1-substituted diazen-1-ium-1,2-diolates) as nitric oxide donors: Convenient nitric oxide dosage forms Methods in Enzymology. vol Volume 268 1996 Academic Press, New York 281 293 10.1016/s0076-6879(96)68030-6 8782594

[B22] Kroupitski Y Pinto R Brandl MT Belausov E Sela S Interactions of *Salmonella enterica* with lettuce leaves J Appl Microbiol 2009 4 6 1876 1885 10.1111/j.1365-2672.2009.04152.x 19239550

[B23] Kroupitski Y Brandl MT Pinto R Belausov E Tamir-Ariel D Burdman S Sela Saldinger S Identification of *Salmonella enterica* genes with a role in persistence on lettuce leaves during cold storage by Recombinase-based in Vivo Expression Technology Phytopathology 2013 4 4 362 372 10.1094/PHYTO-10-12-0254-FI 23506363

[B24] Landini P Antoniani D Burgess JG Nijland R Molecular mechanisms of compounds affecting bacterial biofilm formation and dispersal Appl Microbiol Biotechnol 2010 4 3 813 823 10.1007/s00253-010-2468-8 20165945

[B25] Maragos CM Morley D Wink DA Dunams TM Saavedra JE Hoffman A Bove AA Isaac L Hrabie JA Keefer LK Complexes of NO with nucleophiles as agents for the controlled biological release of nitric oxide: Vasorelaxant effects J Med Chem 1991 4 11 3242 3247 10.1021/jm00115a013 1956043

[B26] Marvasi M Visscher PT Casillas Martinez L Exopolymeric substances (EPS) from *Bacillus subtilis*: polymers and genes encoding their synthesis FEMS Microbiol Lett 2010 4 1 1 9 10.1111/j.1574-6968.2010.02085.x 20735481

[B27] McDougald D Rice SA Barraud N Steinberg PD Kjelleberg S Should we stay or should we go: mechanisms and ecological consequences for biofilm dispersal Nat Rev Micro 2012 4 1 39 50 10.1038/nrmicro2695 22120588

[B28] Merritt JH Kadouri DE O’Toole GA Growing and analyzing static biofilms Curr Protoc Microbiol 2005 4 Unit 1B 1 doi:10.1002/9780471729259.mc01b01s00 18770545 10.1002/9780471729259.mc01b01s00 PMC4568995

[B29] Miller C McMullin B Ghaffari A Stenzler A Pick N Roscoe D Ghahary A Road J Av-Gay Y Gaseous nitric oxide bactericidal activity retained during intermittent high-dose short duration exposure Nitric Oxide 2009 4 1 16 23 10.1016/j.niox.2008.08.002 18789393

[B30] Noel JT Arrach N Alagely A McClelland M Teplitski M Specific responses of *Salmonella enterica* to tomato varieties and fruit ripeness identified by in vivo expression technology PLoS One 2010 4 8 e12406 10.1371/journal.pone.0012406 20824208 PMC2930847

[B31] O’Toole GA Kolter R Initiation of biofilm formation in *Pseudomonas fluorescens* WCS365 proceeds via multiple, convergent signalling pathways: a genetic analysis Mol Microbiol 1998 4 3 449 461 10.1046/j.1365-2958.1998.00797.x 9632250

[B32] Petrova OE Sauer K Dispersion by *Pseudomonas aeruginosa* requires an unusual posttranslational modification of BdlA Proc Natl Acad Sci U S A 2012 4 41 16690 16695 10.1073/pnas.1207832109 23012421 PMC3478618

[B33] Plate L Marletta MA Nitric oxide modulates bacterial biofilm formation through a multicomponent cyclic-di-GMP signaling network Mol Cell 2012 4 4 449 460 10.1016/j.molcel.2012.03.023 22542454 PMC3361614

[B34] Ramamurthi A Lewis RS Measurement and modeling of nitric oxide release rates for nitric oxide donors Chem Res Toxicol 1997 4 4 408 413 10.1021/tx960183w 9114977

[B35] Regev-Shoshani G Ko M Miller C Av-Gay Y Slow release of nitric oxide from charged catheters and its effect on biofilm formation by *Escherichia coli* Antimicrob Agents Chemother 2010 4 1 273 279 10.1128/AAC.00511-09 19884372 PMC2798533

[B36] Richardson AR Payne EC Younger N Karlinsey JE Thomas VC Becker LA Navarre WW Castor ME Libby SJ Fang FC Multiple targets of nitric oxide in the tricarboxylic acid cycle of *Salmonella enterica* serovar typhimurium Cell Host Microbe 2011 4 1 33 43 10.1016/j.chom.2011.06.004 21767810 PMC3142370

[B37] Rosenkranz B Winkelmann BR Parnham MJ Clinical pharmacokinetics of molsidomine Clin Pharmacokinet 1996 4 5 372 384 10.2165/00003088-199630050-00004 8743336

[B38] Santiviago CA Reynolds MM Porwollik S Choi S-H Long F Andrews-Polymenis HL McClelland M Analysis of pools of targeted *Salmonella* deletion mutants identifies novel genes affecting fitness during competitive infection in mice PLoS Pathog 2009 4 7 e1000477 10.1371/journal.ppat.1000477 19578432 PMC2698986

[B39] Shi X Zhu X Biofilm formation and food safety in food industries Trend Food Sci Technol 2009 4 9 407 413 doi:http://dx.doi.org/10.1016/j.tifs.2009.01.054 10.1016/j.tifs.2009.01.054

[B40] Simontacchi M García-Mata C Bartoli CG Santa-María GE Lamattina L Nitric oxide as a key component in hormone-regulated processes Plant Cell Rep 2013 4 6 853 866 10.1007/s00299-013-1434-1 23584547

[B41] Slomberg DL Lu Y Broadnax AD Hunter RA Carpenter AW Schoenfisch MH Role of size and shape on biofilm eradication for nitric oxide-releasing silica nanoparticles ACS Appl Mater Interfaces 2013 4 19 10.1021/am402618w 24006838

[B42] Solano C Garcia B Valle J Berasain C Ghigo JM Gamazo C Lasa I Genetic analysis of *Salmonella enteritidis* biofilm formation: critical role of cellulose Mol Microbiol 2002 4 3 793 808 10.1046/j.1365-2958.2002.02802.x 11929533

[B43] Teplitski M Al-Agely A Ahmer BMM Contribution of the SirA regulon to biofilm formation in *Salmonella enterica* serovar Typhimurium Microbiology 2006 4 11 3411 3424 10.1099/mic.0.29118-0 17074910

[B44] Thompson SE Callow ME Callow JA The effects of nitric oxide in settlement and adhesion of zoospores of the green alga *Ulva* Biofouling 2010 4 2 167 178 10.1080/08927010903402420 19927239

[B45] Van Alst NE Picardo KF Iglewski BH Haidaris CG Nitrate sensing and metabolism modulate motility, biofilm formation, and virulence in *Pseudomonas aeruginosa* Infect Immun 2007 4 8 3780 3790 10.1128/IAI.00201-07 17526746 PMC1952006

[B46] Wang PG Cai TB Taniguchi N Nitric Oxide Donors 2005 Wiley-VCH Verlag GmbH & Co, KGaA

[B47] Wingender J Neu TR Flemming HC Microbial Extracellular Polymeric Substances: Characterization, Structure, and Function 1999 Springer, New York

[B48] Xiong Y Liu Y Biological control of microbial attachment: a promising alternative for mitigating membrane biofouling Appl Microbiol Biotechnol 2010 4 3 825 837 10.1007/s00253-010-2463-0 20169341

[B49] Zhang L Mah TF Involvement of a novel efflux system in biofilm-specific resistance to antibiotics J Bacteriol 2008 4 13 4447 4452 10.1128/JB.01655-07 18469108 PMC2446775

