# Supplementary material for: Systematic analysis of the ability of Nitric Oxide donors to dislodge biofilms formed by Salmonella enterica and Escherichia coli O157:H7
Source: AMB Express. 2014 Jun 5;4:42. doi: 10.1186/s13568-014-0042-y (PMC4070026; doi:10.1186/s13568-014-0042-y)
Supplement: Additional file 1: Figure S1. — Biofilm formation on different plastics by Salmonella and E. coli O157:H7. Biofilms were established on polypropylene and polystyrene surfaces in CFA medium for 24 hrs and stained with 1% crystal violet and then washed. The absorbed crystal violet and the biofilm were dissolved in 33% acetic acid and A590 was measured with a spectrophotometer. Error bars represent standard error. Significant different means are displayed with different letters. Figure S2. Luminescence of Salmonella 14028 pTIM2442 upon exposure to molsidomine. General metabolic state of the cells was assessed using the redox-coupled FMNH2/Luciferase produced by a S. Typhimurium ATCC14028 strain harboring high copy number plasmid in which the luxCDABE operon is under the phage λ promoter (pTIM2442). Concentrations of molsidomine to which cultures of Salmonella 14028 pTIM2442 were exposed are listed on the figure. Error bars represent the standard error of 12 replicas. Table S1. Fluorescence of the Salmonella cells detached by molsidomine treatment Measurement of Salmonella 14028 planktonic cells detached from preformed biofilm during treatment with molsidomine. polypropylene, *Statistically significant effects (p<0.05) of molsidomine treatment. Table S2. The complete set of experiments on the effect of the NO donors on preformed biofilm after 24 hours of contact time. *Statistically significant effects (p<0.05) of the NO donor treatments. n.s., not statistically significant biofilm dispersion. MOL, Molsidomine; M1555, MAHMA NONOate; S150, SPERMINE NONOate; D5431, diethylamine NONOate diethylammonium salt; D184, diethylamine NONOate sodium; S8432 sulfo NONOate disodium salt. Table S3. The complete set of experiments on the effect of the NO donors on preformed biofilm after 6 hours of contact time. *Statistically significant effects (p<0.05) of the NO donor treatments. n.s., not statistically significant biofilm dispersion. MOL, Molsidomine; M1555, MAHMA NONOate; S150, SPERMINE NONOate; D5431, diethylam [file s13568-014-0042-y-S1.pdf]

Supplementary File

Article title: Systematic analysis of the ability of Nitric Oxide donors to dislodge biofilms formed by *Salmonella enterica* and *Escherichia coli* O157:H7

Journal Name: AMB Express

Authors and Affiliations: Massimiliano Marvasi[1], Charles Chen[2], Manuel Carrazana[2], Ian A. Durie[1] and Max Teplitski[1*]
[1] Soil and Water Science Department, University of Florida-IFAS
[2] Microbiology and Cell Science Department, University of Florida-IFAS

*Corresponding author. Email address: maxtep@ufl.edu; phone: (352) 273-8189
Mailing address: Cancer and Genetics Research Center, 2033 Mowry Road, Genetics Institute, Gainesville, FL, USA 32611

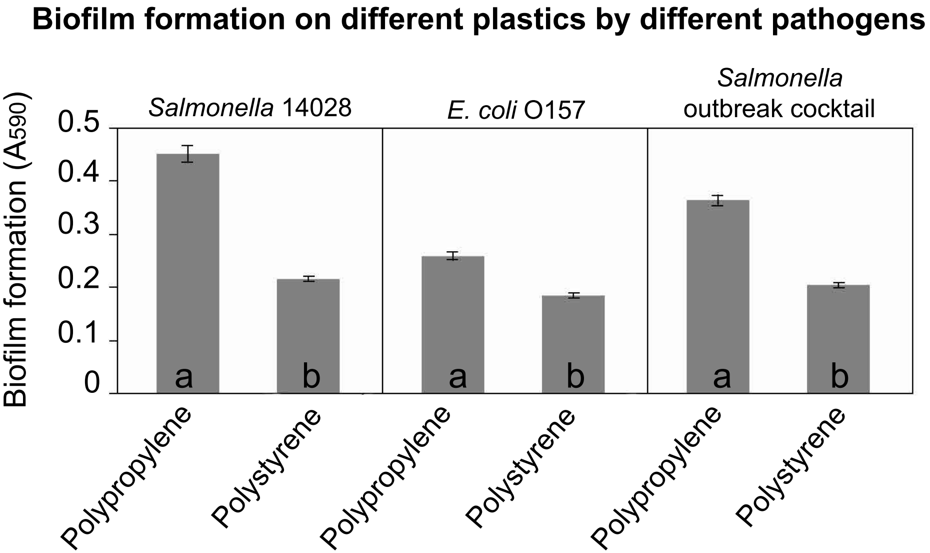

Biofilm formation on different plastics by different pathogens
Salmonella 14028
E. coli O157
Salmonella outbreak cocktail
Biofilm formation (A590)
0.5
0.4
0.3
0.2
0.1
0
a
b
a
b
a
b
Polypropylene
Polystyrene
Polypropylene
Polystyrene
Polypropylene
Polystyrene

Supplemental Material - Figure S1. Biofilm formation on different plastics by *Salmonella* and *E. coli* O157:H7. Biofilms were established on polypropylene and polystyrene surfaces in CFA medium for 24 hrs and stained with 1 % crystal violet and then washed. The absorbed crystal violet and the biofilm were dissolved in 33% acetic acid and A590 was measured with a spectrophotometer. Error bars represent standard error. Significant different means are displayed with different letters.

Luminescence of *Salmonella* 14028 pTIM2442 after molsidomine treatment

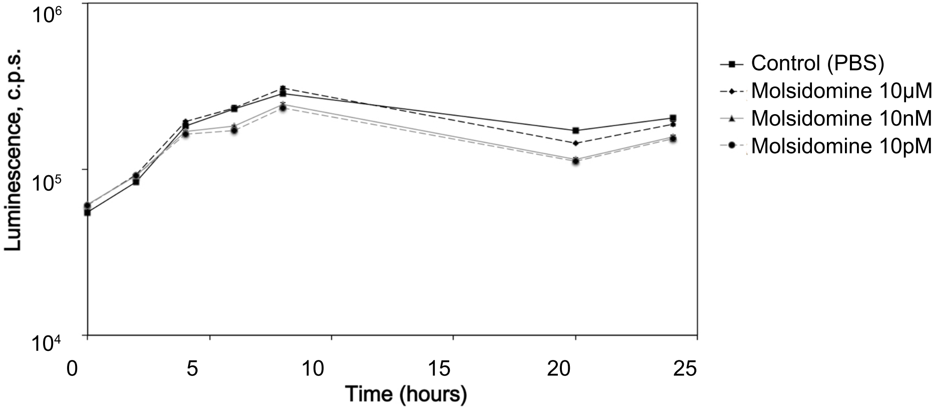

Supplemental Material – Figure S2. Luminescence of *Salmonella* 14028 pTIM2442 upon exposure to molsidomine. General metabolic state of the cells was assessed using the redox-coupled $FMNH_2$/Luciferase produced by a *S.* Typhimurium ATCC14028 strain harboring high copy number plasmid in which the *luxCDABE* operon is under the phage λ promoter (pTIM2442). Concentrations of molsidomine to which cultures of *Salmonella* 14028 pTIM2442 were exposed are listed on the figure. Error bars represent the standard error of 12 replicas.

Supplementary Table S1. Fluorescence of the *Salmonella* cells detached by molsidomine treatment

Measurement of *Salmonella* 14028 planktonic cells detached from preformed biofilm during treatment with molsidomine. polypropylene

Conc.	Log (Fluorescence 485nm/535nm)			P<0.05
	0 hours	3 hours	6 hours	
Biofilms formed on polypropylene				
0	4.335 ± 0.065	n.a.	4.450 ± 0.177	0.5492
10 μM	4.307 ± 0.067	4.846 ± 0.054	4.466 ± 0.080	<.0001*
10 nM	4.275 ± 0.055	4.780 ± 0.050	4.753 ± 0.075	<.0001*
10 pM	4.355 ± 0.064	4.815 ± 0.125	5.002 ± 0.144	0.0024*

*Statistically significant effects ($p<0.05$) of molsidomine treatment.

Supplementary Table S2. The complete set of experiments on the effect of the NO donors on preformed biofilm after 24 hours of contact time.

NOD	Material	Strain	Incubation temperature	Prob>F (p=0.05)
D184	Polypropylene	*Salmonella* 14028	4°C	<0.0001
D184	Polypropylene	*Salmonella* 14028	22°C	n.s.
D184	Polypropylene	*E.coli* O157:H7	4°C	n.s.
D184	Polypropylene	*E.coli* O157:H7	22°C	n.s.
D184	Polypropylene	*Salmonella* cocktail	4°C	n.s.
D184	Polypropylene	*Salmonella* cocktail	22°C	<0.0001
D5431	Polypropylene	*Salmonella* 14028	4°C	<0.0001
D5431	Polypropylene	*Salmonella* 14028	22°C	<0.0001
D5431	Polypropylene	*E.coli* O157:H7	4°C	0.0004
D5431	Polypropylene	*E.coli* O157:H7	22°C	<0.0001
D5431	Polypropylene	*Salmonella* cocktail	4°C	<0.0001
D5431	Polypropylene	*Salmonella* cocktail	22°C	<0.0001
M1555	Polypropylene	*Salmonella* 14028	4°C	n.s.
M1555	Polypropylene	*Salmonella* 14028	22°C	0.0049
M1555	Polypropylene	*E.coli* O157:H7	4°C	n.s.
M1555	Polypropylene	*E.coli* O157:H7	22°C	<0.0001
M1555	Polypropylene	*Salmonella* cocktail	4°C	0.0185
M1555	Polypropylene	*Salmonella* cocktail	22°C	<0.0001
MOL	Polypropylene	*Salmonella* 14028	4°C	<0.0001
MOL	Polypropylene	*Salmonella* 14028	22°C	<0.0001
MOL	Polypropylene	*E.coli* O157:H7	4°C	<0.0001
MOL	Polypropylene	*E.coli* O157:H7	22°C	n.s.
MOL	Polypropylene	*Salmonella* cocktail	4°C	0.0001
MOL	Polypropylene	*Salmonella* cocktail	22°C	<0.0001
D184	Polystyrene	*Salmonella* 14028	4°C	n.s.
D184	Polystyrene	*Salmonella* 14028	22°C	n.s.
D184	Polystyrene	*E.coli* O157:H7	4°C	n.s.
D184	Polystyrene	*E.coli* O157:H7	22°C	n.s.
D184	Polystyrene	*Salmonella* cocktail	4°C	<0.0001
D184	Polystyrene	*Salmonella* cocktail	22°C	<0.0001
D184	Polystyrene	*Salmonella* 14028	4°C	<0.0001
D5431	Polystyrene	*Salmonella* 14028	22°C	0.0006
D5431	Polystyrene	*E.coli* O157:H7	4°C	0.0001
D5431	Polystyrene	*E.coli* O157:H7	22°C	n.s.
D5431	Polystyrene	*Salmonella* cocktail	4°C	<0.0001
D5431	Polystyrene	*Salmonella* cocktail	22°C	<0.0001
M1555	Polystyrene	*Salmonella* 14028	4°C	0.0229
M1555	Polystyrene	*Salmonella* 14028	22°C	<0.0001
M1555	Polystyrene	*E.coli* O157:H7	4°C	n.s.
M1555	Polystyrene	*E.coli* O157:H7	22°C	n.s.
M1555	Polystyrene	*Salmonella* cocktail	4°C	<0.0001

M1555	Polystyrene	*Salmonella* cocktail	22°C	<0.0001
MOL	Polystyrene	*Salmonella* 14028	4°C	0.0465
MOL	Polystyrene	*Salmonella* 14028	22°C	0.0078
MOL	Polystyrene	*E.coli* O157:H7	4°C	n.s.
MOL	Polystyrene	*E.coli* O157:H7	22°C	n.s.
MOL	Polystyrene	*Salmonella* cocktail	4°C	<0.0001
MOL	Polystyrene	*Salmonella* cocktail	22°C	<0.0001

* Statistically significant effects (p<0.05) of the NO donor treatments. n.s., not statistically significant biofilm dispersion. MOL, Molsidomine; M1555, MAHMA NONOate; S150, SPERMINE NONOate; D5431, diethylamine NONOate diethylammonium salt; D184, diethylamine NONOate sodium; S8432 sulfo NONOate disodium salt.

Supplementary Table S3. The complete set of experiments on the effect of the NO donors on preformed biofilm after 6 hours of contact time.

NO donor	Material	Strain	Incubation temperature	Prob>F*
D184	Polypropylene	*Salmonella* 14028	4°C	<0.0001
D184	Polypropylene	*Salmonella* 14028	22°C	n.s.
D184	Polypropylene	*E.coli* O157:H7	4°C	n.s.
D184	Polypropylene	*E.coli* O157:H7	22°C	0.0551
D184	Polypropylene	*Salmonella* cocktail	4°C	n.s.
D184	Polypropylene	*Salmonella* cocktail	22°C	<0.0001
D5431	Polypropylene	*Salmonella* 14028	4°C	<0.0001
D5431	Polypropylene	*Salmonella* 14028	22°C	<0.0001
D5431	Polypropylene	*E.coli* O157:H7	4°C	0.0004
D5431	Polypropylene	*E.coli* O157:H7	22°C	<0.0001
D5431	Polypropylene	*Salmonella* cocktail	4°C	<0.0001
D5431	Polypropylene	*Salmonella* cocktail	22°C	<0.0001
M1555	Polypropylene	*Salmonella* 14028	4°C	n.s.
M1555	Polypropylene	*Salmonella* 14028	22°C	0.0049
M1555	Polypropylene	*E.coli* O157:H7	4°C	n.s.
M1555	Polypropylene	*E.coli* O157:H7	22°C	<0.0001
M1555	Polypropylene	*Salmonella* cocktail	4°C	n.s.
M1555	Polypropylene	*Salmonella* cocktail	22°C	<0.0001
MOL	Polypropylene	*Salmonella* 14028	4°C	<0.0001
MOL	Polypropylene	*Salmonella* 14028	22°C	<0.0001
MOL	Polypropylene	*E.coli* O157:H7	4°C	<0.0001
MOL	Polypropylene	*E.coli* O157:H7	22°C	n.s.
MOL	Polypropylene	*Salmonella* cocktail	4°C	0.0001
MOL	Polypropylene	*Salmonella* cocktail	22°C	<0.0001
S150	Polypropylene	*Salmonella* 14028	4°C	n.s.
S150	Polypropylene	*Salmonella* 14028	22°C	n.s.
S150	Polypropylene	*E.coli* O157:H7	4°C	0.0273
S150	Polypropylene	*E.coli* O157:H7	22°C	0.0486
S150	Polypropylene	*Salmonella* cocktail	4°C	0.0036
S150	Polypropylene	*Salmonella* cocktail	22°C	0.0003
D184	Polystyrene	*Salmonella* 14028	4°C	n.s.
D184	Polystyrene	*Salmonella* 14028	22°C	n.s.
D184	Polystyrene	*E.coli* O157:H7	4°C	n.s.
D184	Polystyrene	*E.coli* O157:H7	22°C	n.s.
D184	Polystyrene	*Salmonella* cocktail	4°C	<0.0001
D184	Polystyrene	*Salmonella* cocktail	22°C	<0.0001
D5431	Polystyrene	*Salmonella* 14028	4°C	<0.0001
D5431	Polystyrene	*Salmonella* 14028	22°C	0.0006
D5431	Polystyrene	*E.coli* O157:H7	4°C	0.0001
D5431	Polystyrene	*E.coli* O157:H7	22°C	n.s.

D5431	Polystyrene	*Salmonella* cocktail	4°C	<0.0001
D5431	Polystyrene	*Salmonella* cocktail	22°C	<0.0001
M1555	Polystyrene	*Salmonella* 14028	4°C	0.0229
M1555	Polystyrene	*Salmonella* 14028	22°C	<0.0001
M1555	Polystyrene	*E.coli* O157:H7	4°C	n.s.
M1555	Polystyrene	*E.coli* O157:H7	22°C	n.s.
M1555	Polystyrene	*Salmonella* cocktail	4°C	0.0184
M1555	Polystyrene	*Salmonella* cocktail	22°C	<0.0001
MOL	Polystyrene	*Salmonella* 14028	4°C	<0.0001
MOL	Polystyrene	*Salmonella* 14028	22°C	0.0078
MOL	Polystyrene	*E.coli* O157:H7	4°C	n.s.
MOL	Polystyrene	*E.coli* O157:H7	22°C	n.s.
MOL	Polystyrene	*Salmonella* cocktail	4°C	<0.0001
MOL	Polystyrene	*Salmonella* cocktail	22°C	<0.0001
S150	Polystyrene	*Salmonella* 14028	4°C	0.0418
S150	Polystyrene	*Salmonella* 14028	22°C	n.s.
S150	Polystyrene	*E.coli* O157:H7	4°C	n.s.
S150	Polystyrene	*E.coli* O157:H7	22°C	n.s.
S150	Polystyrene	*Salmonella* cocktail	4°C	n.s.
S150	Polystyrene	*Salmonella* cocktail	22°C	0.0183

* Statistically significant effects ($p<0.05$) of the NO donor treatments. n.s., not statistically significant biofilm dispersion. MOL, Molsidomine; M1555, MAHMA NONOate; S150, SPERMINE NONOate; D5431, diethylamine NONOate diethylammonium salt; D184, diethylamine NONOate sodium; S8432 sulfo NONOate disodium salt.
